# Drotrecogin alfa (activated): real-life use and outcomes for the UK

**DOI:** 10.1186/cc6879

**Published:** 2008-04-22

**Authors:** Kathryn M Rowan, Catherine A Welch, Emma North, David A Harrison

**Affiliations:** 1 Intensive Care National Audit & Research Centre, Tavistock House, Tavistock Square, London WC1H 9HR, UK

## Abstract

**Introduction:**

In March 2001, the results of the Recombinant Human Activated Protein C Worldwide Evaluation in Severe Sepsis (PROWESS) study were published, which indicated a 6.1% absolute reduction in 28-day mortality. Drotrecogin alfa (activated; DrotAA) was subsequently approved for use in patients with severe sepsis.

**Methods:**

In December 2002, critical care units in England, Wales and Northern Ireland were invited to participate in an audit of DrotAA. Data for each infusion of DrotAA were linked to case mix and outcome data from a national audit. Use of DrotAA was described and a nonrandomized comparison of effectiveness was conducted.

**Results:**

1,292 infusions of DrotAA were recorded in 112 units; 61% commenced during the first 24 hours in the unit. The majority (77%) of patients had three or more organs failing; lung (42%) and abdomen (40%) were the most common primary sites of infection. Crude hospital mortality was high (45%); at 28 days, only 18% had left acute hospital and 19% were still in the unit. For 30%, the full 96-hour infusion was not completed; 24% of infusions were interrupted; 8.1% experienced one or more serious adverse events, of which 77% were serious bleeding events. Of eight relative risks estimated from individually-matched (0.75 to 0.85) and propensity-matched (0.82 to 0.90) controls, seven were consistent with the results of PROWESS. Restricting the analysis to patients receiving DrotAA during the first 24 hours resulted in larger treatment effects (relative risks 0.62 to 0.81). For all matches, similar patterns were seen across subgroups. No effect of DrotAA was seen for two organs failing or lower severity scores, compared with a significant mortality reduction for three or more organs failing or higher severity scores.

**Conclusion:**

Use of DrotAA was approximately one in 16 for admissions meeting the definition for severe sepsis and with two or more organs failing. Patients receiving DrotAA were younger and more severely ill but were less likely to have serious conditions in their past medical history. Nonrandomized estimates for the effectiveness of DrotAA were consistent with the findings of PROWESS. DrotAA appeared not to be effective in patients with less severe disease.

## Introduction

In March 2001 the results of the Recombinant Human Activated Protein C Worldwide Evaluation in Severe Sepsis (PROWESS) study were published [1], indicating a 6.1% (95% confidence interval 1.9% to 10.4%) absolute reduction (19.4% relative reduction) in 28-day mortality. Drotrecogin alfa (activated; DrotAA; Xigris^®^, Eli Lilly and Company, Indianapolis, Indiana, USA) was subsequently approved for use in patients with severe sepsis by the US Food and Drug Administration (FDA) in October 2001 and by the European Medicines Evaluation Agency in August 2002.

During this period, controversy arose regarding the effectiveness of DrotAA, stemming from a number of issues. First, controversy surrounded the original PROWESS study. Specifically, the protocol and cell bank were changed during recruitment; it was stopped early (showing benefit); patients were only followed to 28 days (with survivors still in intensive care or hospital); subgroup analyses indicated that benefit existed solely for high-risk patients; and use appeared to be associated with serious bleeding. Second, controversy surrounded the approval process. The US FDA advisory panel was evenly split (10 in favour and 10 against) [2], with four of the dissenting FDA advisors outlining their concerns in an opinion piece [3], which was subsequently answered by the FDA [4] and PROWESS investigators [5]. In addition, the European license was granted under 'exceptional circumstances', indicating that data on efficacy were not comprehensive and placing a requirement for annual reassessment. Third, controversy surrounded postapproval activities by Eli Lilly and Company, particularly relating to the financial sponsorship for development of clinical guidelines by the Surviving Sepsis Campaign [6].

These areas of controversy, combined with the fact that no UK centres had participated in the original PROWESS study and the high cost of the drug (the average cost per 96-hour infusion was £4,905), led to considerable uncertainty and debate surrounding the effectiveness of DrotAA and its use in the UK. In addition, epidemiological data suggested that 29% of all admissions to adult, general critical care units in the UK (about 31,000 patients per year) had severe sepsis during the first 24 hours in the critical care unit (82% had two or more organs failing), and 45% (about 15,000 patients per year) died before discharge from the acute hospital [7]. The mean length of stay in the critical care unit for an admission with severe sepsis was 8 days, as compared with around 3.5 days for other admissions. Admissions with severe sepsis accounted for almost half of all critical care unit bed-days, at a total cost of around £425 million per annum.

A combination of the controversy, the debate and the burden that severe sepsis imposes on UK critical care prompted the Intensive Care National Audit & Research Centre (ICNARC), following European licensing of DrotAA, to initiate a large, multicentre audit of its use and outcomes. The aims of this audit were as follows: to monitor the real-life use of DrotAA and subsequent outcomes; to undertake a rigorous, nonrandomized evaluation of the effectiveness of DrotAA, by linking the data on DrotAA to the ongoing outcome audit for all admissions to adult, general critical care units; and to compare our results with those from the PROWESS study.

## Materials and methods

### Case Mix Programme

The Case Mix Programme (CMP) is a national comparative audit of adult, general critical care units (including intensive care and combined intensive care and high dependency units) in England, Wales and Northern Ireland, and is coordinated by ICNARC. Approximately 75% of units participate, and so it provides highly representative data. Prospective, raw clinical data are abstracted retrospectively, in accordance with precise rules and definitions, by trained, local data collectors, and undergo extensive validation, both locally and centrally. CMP data collection and validation processes were previously reported [8] and have been independently assessed to be of high quality [9].

CMP data collection is restricted to first 24-hour case mix (age, acute severity, past medical history, surgical status and reason for admission) and outcomes (unit/acute hospital discharge status) for the purposes of outcome audit, employing accurate risk prediction models [10]. Support for the collection and use of patient-identifiable data without consent was obtained under Section 60 of the UK Health and Social Care Act of 2001 (approval number: PIAG 2-10[f]/2005).

### Audit of DrotAA

In December 2002 all units participating in the CMP were invited to participate in an audit of DrotAA. Participating units completed a one-page data collection form for each patient admitted who received DrotAA during their unit stay (Additional data file 1). Details of the infusion, the underlying infection and serious adverse events were requested. Completed forms were sent centrally for data entry and validation. In addition, quarterly confirmation reports for DrotAA infusions were signed off as accurate by a senior doctor in each unit. CMP and DrotAA data were linked in order to provide demographics, case mix and outcomes from the CMP.

### Severe sepsis

Those patients admitted with severe sepsis or who developed severe sepsis during the first 24 hours in the critical care unit were identified using criteria derived from PROWESS [7,11]. Briefly, severe sepsis was defined as evidence of infection plus three or more systemic inflammatory response syndrome criteria [12] and at least one organ failing (cardiovascular, respiratory, renal, haematological, or metabolic) during the first 24 hours.

### Sample size calculation

Sample size was calculated to reproduce the original intended sample size for PROWESS (1,140 patients receiving DrotAA), in order to give 90% power to detect (*P *< 0.05) a relative risk reduction of 15%, as compared with the 20% relative risk reduction observed in PROWESS. A statistical analysis plan was agreed *a priori*.

### Use of DrotAA

Rate of use of DrotAA, both in patients with severe sepsis and across critical care units, was calculated. Case mix and outcomes for patients receiving DrotAA were described. Details relating to the infusion of DrotAA, including time to initiation, duration and interruptions, were described. Type, site and characteristics of patients having serious adverse events were also described.

### Nonrandomized comparison

Matched cohort analyses were performed using two statistical approaches: individual matching on patient factors and propensity matching.

Each patient admitted who met the PROWESS-derived definition for severe sepsis plus two or more organs failing during the first 24 hours in the critical care unit, and in whom an infusion of DrotAA was commenced, was matched one-to-one with four pools of control patients:

1. an historic admission from the same unit, from January 2000 onward and before the European Medicines Evaluation Agency approval of DrotAA on 22 August 2002 (excluding admissions, from March 2001 onwards, in units included in Extended Evaluation of Recombinant Activated Protein C [ENHANCE]) [13];

2. a concurrent admission from the same unit, during the audit (January 2004 onward);

3. a concurrent admission from a unit that participated in the audit but did not have local approval for use and did not use DrotAA at any time; and

4. a concurrent admission from a unit that used DrotAA but before the first use in that unit.

All potential control patients met the PROWESS-derived definition for severe sepsis and two or more organs failing but did not receive DrotAA. Readmissions to the critical care unit of the same patient during the same hospital stay were excluded. For each admission receiving DrotAA, matched control patients were selected from among those patients who remained in the unit, alive and receiving active treatment, at the time when the infusion of DrotAA was commenced.

Individual matching was based on the following: admission source (theatre-elective, theatre-emergency, critical care transfer, ward, emergency department, or other hospital); number of organs failing; combined presence of renal and cardiovascular failures (earlier epidemiological analysis of CMP data indicated significantly worse outcomes for severe sepsis with this combination) [11]; ICNARC physiology score [10] (nearest in absolute value to a maximum difference of 10 points); age (nearest in absolute value, out of those with the closest match on physiology score, to a maximum difference of 10 years); and critical care unit (matches 1 and 2 [from the numbered list above]) or hospital type (university, university-affiliated or non-university; matches 3 and 4).

A propensity model for receiving DrotAA was built using multilevel logistic regression, including patient and unit factors. Patient factors included were as follows: age and ICNARC physiology score (fitted as smoothed functions using restricted cubic splines); sex; admission source; organs failing; severe conditions in the past medical history; and body system for the primary reason for admission. Unit factors included were hospital type, number of beds in unit and local approval for DrotAA. The discrimination of the propensity model was assessed by determining the area under the receiver operating characteristic curve, and the overall fit by determining the pseudo-R^2 ^statistic (proportion of log-likelihood explained by the model). A propensity score (predicted log-odds of receiving DrotAA) was calculated from the model, based on patient factors only. Each patient in whom an infusion of DrotAA was commenced was matched one-to-one with patients from the same four control pools as for the individual matching, up to a maximum difference of 0.5 in the predicted log-odds.

Resulting matched cohort data (both individual and propensity matching) were analyzed using conditional fixed-effects cross-sectional Poisson regression models with bootstrapped standard errors [14]. Regression models were adjusted for the ICNARC model predicted log-odds of hospital mortality, in order to account for residual differences within the matched pairs [10].

Subgroup analyses were defined, *a priori*, based on PROWESS subgroups [15,16]. Significant differences were evaluated by testing for interactions between the subgroup categories and treatment effect in the regression model. Subgroups were as follows: age (quartiles), sex, number of organs failing (2, 3 or 4+), Acute Physiology and Chronic Health Evaluation II score (<19, 20 to 24, 25 to 29, 30+) and ICNARC physiology score (quartiles).

Two sensitivity analyses were performed. The first analysis included only those patients in whom an infusion of DrotAA was commenced within 24 hours after admission (and matched control patients), representing matched pairs in which physiology used for matching most closely resembled that at the time of infusion. The second analysis included only those matched pairs in which the ICNARC physiology score was within 5 points, representing closer matches.

All analyses were performed using Stata 9.2 (StataCorp LP, College Station, TX, USA).

## Results

### Participation

Of 197 units invited to participate in the audit 161 (82%) responded, and of these 133 (83%) agreed. Thirteen units withdrew, or were lapsed, because of noncompletion of study documentation, and a further eight units were excluded because no CMP data were available. Overall, 112 units (57% of all units invited) actively participated (Additional data file 2), of which 104 units commenced at least one infusion of DrotAA. Participating units were representative of all units in the CMP in terms of reported hospital type (22% university, 20% university affiliated and 57% non-university), and reported size of unit (median [interquartile range] beds 7.5 [6 to 10]), which in turn is representative of all units in England, Wales and Northern Ireland.

### Use of DrotAA

A total of 1,292 infusions of DrotAA were recorded – an average of 5.3 infusions per unit per year. Patients receiving DrotAA were predominantly nonsurgical (with surgical defined as admission source theatre and recovery) and were highly likely to be ventilated during the first 24 hours (Table 1). In three-quarters of patients, three or more organs were in failure during the first 24 hours; renal and haematological were the least likely. Lung and abdomen were the most commonly reported primary sites of infection. Crude mortality was high, and at 28 days only 17.5% (27.3% of survivors) had left the acute hospital; median length of acute hospital stay for survivors was 44 days (Table 1).

**Table 1:** Characteristics of patients receiving DrotAA compared with control patients

Characteristic	All admissions receiving DrotAA (n = 1,292)	Admissions with severe sepsis and ≥2 organ systems failing receiving DrotAA (N = 1,079)	Admissions with severe sepsis and ≥2 organ systems failing not receiving DrotAA (n = 15,939)
Age (years; mean ± SD)	58.8 ± 16.0	59.1 ± 16.1	63.3 ± 17.2
Sex (*n *[%])			
Female	638 (49.4)	535 (49.6)	7,333 (46.0)
Male	654 (50.6)	544 (50.4)	8,604 (54.0)
Mechanical ventilation on admission or during first 24 hours of stay in critical care (*n *[%])	1,163 (90.0)	998 (92.5)	12,060 (75.7)
APACHE II score (mean ± SD)			
Acute physiology score	18.2 (6.5)	18.8 (6.4)	16.0 (6.5)
Score	21.9 (6.9)	22.6 (6.7)	20.7 (7.2)
ICNARC model physiology score (mean ± SD)	29.1 (9.0)	30.4 (8.4)	24.9 (9.4)
Serious conditions in past medical history^a ^(*n *[%])			
Liver	10 (0.8)	9 (0.8)	384 (2.4)
Cardiovascular	5 (0.4)	5 (0.5)	263 (1.7)
Respiratory	23 (1.8)	23 (2.1)	630 (4.0)
Renal	14 (1.1)	12 (1.1)	315 (2.0)
Immunosuppressed	92 (7.1)	78 (7.2)	1,545 (9.7)
Admission type^b ^(*n *[%])			
Medical	939 (72.7)	789 (73.1)	11,375 (71.4)
Elective surgical	56 (4.3)	32 (3.0)	819 (5.1)
Emergency surgical	297 (23.0)	258 (23.9)	3,745 (23.5)
Organ systems failing during first 24 hours of stay in critical care (*n *[%])			
Cardiovascular	1,235 (95.6)	1,070 (99.2)	15,136 (95.0)
Respiratory	1,166 (90.3)	1,005 (93.1)	13,722 (86.1)
Renal	480 (37.7)	427 (40.0)	4,274 (27.1)
Haematological	229 (17.7)	199 (18.4)	2,290 (14.4)
Metabolic acidosis	990 (76.6)	872 (80.8)	9,674 (60.7)
Number of organ systems failing during first 24 hours of stay in critical care (*n *[%])			
<2	60 (4.7)	N/A	N/A
2	238 (18.4)	198 (18.4)	6,809 (42.7)
3	498 (38.5)	433 (40.1)	5,664 (35.5)
4	398 (30.8)	362 (33.6)	2,844 (17.8)
5	98 (7.6)	86 (8.0)	622 (3.9)
Primary site of infection^c ^(*n *[%])			
Lung	495 (41.9)	423 (42.8)	N/R
Abdomen	478 (40.4)	390 (39.5)	N/R
Urinary tract	57 (4.8)	51 (5.2)	N/R
Other	158 (13.4)	131 (13.3)	N/R
Positive blood culture^d ^(*n *[%])	438 (40.3)	381 (42.1)	N/R
Organisms cultured (*n *[%])			
Any	814 (64.6)	692 (65.8)	N/R
Gram-negative	399 (49.0)	331 (47.8)	N/R
Gram-positive	437 (53.7)	382 (55.2)	N/R
Fungus	101 (12.4)	81 (11.7)	N/R
Deaths within 96 hours of unit admission (*n *[%])	166 (12.9)	151 (14.0)	3,315 (20.8)
Mortality (deaths [%])			
Unit	449 (34.8)	385 (35.7)	5,404 (33.9)
Hospital^e^	567 (45.0)	484 (45.4)	7,438 (46.7)
Location at 28-days (*n *[%])			
Died	463 (35.8)	398 (36.9)	6,541 (41.0)
Unit	240 (18.6)	177 (16.4)	888 (5.6)
Hospital	363 (28.1)	312 (28.9)	3,865 (24.3)
Out of Hospital^e^	226 (17.5)	192 (17.8)	4,645 (29.1)
Unit length of stay (days; median [IQR])			
Unit survivors	14.2 (8.1–24.9)	13.8 (8.0–23.2)	5.0 (2.2–11.0)
Unit nonsurvivors	7.2 (2.3–16.3)	6.4 (2.0–15.6)	2.5 (1.0–7.4)
Hospital length of stay (days; median [IQR])^f^			
Hospital survivors	44 (25–69)	44 (24–66)	29 (16–52)
Hospital nonsurvivors	15 (6–31)	14 (5–28)	11 (4–25)
Destination following discharge from hospital housing critical care unit (*n *[%])			
Another acute hospital	41 (6.5)	29 (5.5)	537 (6.8)
Hospice or equivalent	8 (1.3)	8 (1.5)	40 (0.5)
Long-term institutional care	13 (2.0)	12 (2.3)	126 (1.6)
Rehabilitation unit	43 (6.8)	35 (6.6)	549 (7.0)
Normal residence	531 (83.5)	446 (84.2)	6,652 (84.2)

^a^Fourteen (1.1%) admissions had no evidence to assess past medical history. Evidence defined as follows, during prior 6 months (except those terms highlighted with asterisk, which are not restricted to the preceding 6 months): liver (biopsy proven cirrhosis, portal hypertension, hepatic encephalopathy); cardiovascular (very severe cardiovascular disease [New York Heart Association functional classification IV]); respiratory (severe respiratory disease [shortness of breath with light activity, for example walking 20 m on level ground], home ventilation); renal (chronic renal replacement therapy for irreversible renal disease); immunosuppressed (AIDS*, steroid treatment [daily], radiotherapy, chemotherapy, metastatic disease, acute myelogenous/lymphocytic leukaemia or multiple myeloma, chronic myelogenous/lymphocytic leukaemia, lymphoma, congenital immunohumoral or cellular immune deficiency state*). ^b^Surgical admissions are defined as those admitted directly to the unit from theatre and recovery. ^c^Primary site of infection was not reported for 110 (8.5%) admissions. ^d^A blood culture was reported as not done for 181 (14.0%) admissions. ^e^Discharged alive from acute hospital at or before 28 days. ^f^Excluding readmissions within the same hospital stay. APACHE, Acute Physiology and Chronic Health Evaluation; DrotAA, Drotrecogin alfa (activated); ICNARC, Intensive Care National Audit & Research Centre; IQR, interquartile range; SD, standard deviation.

DrotAA infusions were commenced predominantly during the first 24 hours (60.7% [772/1,271] of whose for whom timing data were available), but nearly one-third of all DrotAA infusions were reported as not completing the full 96 hours; deterioration to death was reported as accounting for half of these and actual or risk for bleeding accounted for a further quarter (Table 2). Infusions were reported as interrupted for almost a quarter of patients, with just over one-tenth for bleeding-related issues. On average, interruptions lasted 5 hours (Table 2).

**Table 2:** Data relating to infusions of DrotAA

Infusion-related data	All admissions receiving DrotAA (n = 1,292)
Time from unit admission to start of infusion (hours; median [IQR])	19.5 (9.3–35.3)
Received complete 96-hour infusion (*n *[%])	896 (69.9)
Reason for not receiving complete infusion (*n *[%])	
Deterioration/treatment withdrawn/died	195 (50.8)
Actual/possible bleeding	91 (23.7)
Patient improved/left ward	34 (8.9)
Other intervention/treatment outside unit	16 (4.2)
Criteria reassessed/incorrect	9 (2.3)
Timing error	8 (2.1)
Other intervention/treatment in unit	5 (1.3)
Macro/micro drug supply issues	4 (1.0)
Infused over shorter time period	1 (0.3)
No reason given/not known	21 (5.5)
Interruption in the infusion (*n *[%])	304 (24.0)
Time from start of infusion to interruption (hours; median [IQR])	24.0 (13.2–49.0)
Duration of interruption (hours; median [IQR])	5 (3–10)
Reason for interruption (*n *[%])	
Lines/catheters/cannula/drain/dressing-inserted/changed/removed/re-sited/fell out	144 (48.0)
To theatre	48 (16.0)
Bleeding related (actual/suspected)	35 (11.7)
Intervention off unit	15 (5.0)
Tracheostomy	15 (5.0)
Intervention on unit	11 (3.7)
In error	10 (3.3)
Macro/micro drug supply issues	10 (3.3)
Patients condition improved/left unit	1 (0.3)
No reason given/not known	11 (3.7)

IQR, interquartile range.

One or more serious adverse events were reported for 104 patients (8.1%). Of these, 80 (76.9%) were serious bleeding events, 10 (9.6%) were thrombotic events and 19 (18.3%) were other events. Of the serious bleeding events (Table 3), gastrointestinal bleeds were reported as accounting for a third, and skin or soft tissue bleeds accounting for one-fifth. Patients with reported serious bleeding events were more likely to have three or more organs failing and were less likely to have the lung as the primary site of infection. Serious bleeding events were associated, unsurprisingly, with higher mortality and longer lengths of stay (Table 3).

**Table 3:** Characteristics of patients experiencing serious bleeding events

Characteristics	Admissions experiencing serious bleeding events (n = 80)
Site of serious bleeding event (*n *[%])	
Gastrointestinal	29 (33.7)
Skin or soft tissue	19 (22.1)
Intra-abdominal	9 (10.5)
Intracranial	7 (8.1)
Intrathoracic	5 (5.8)
Genitourinary	3 (3.5)
Retroperitoneal	0 (0.0)
Other (source identified)^a^	6 (7.0)
Other (source unidentified)	5 (5.8)
Age (years; mean ± SD)	58.5 (14.9)
APACHE II score (mean ± SD)	
Acute physiology score	19.6 (7.5)
Score	22.9 (7.6)
ICNARC model physiology score (mean ± SD)	30.5 (8.3)
Serious conditions in past medical history^b ^(*n *[%])	
Liver	1 (1.3)
Cardiovascular	0 (0.0)
Respiratory	0 (0.0)
Renal	1 (1.3)
Immunosuppressed	4 (5.0)
Admission type^c ^(*n *[%])	
Medical	55 (68.8)
Elective surgical	3 (3.8)
Emergency surgical	22 (27.5)
Organ systems failing during first 24 hours of stay in critical care (*n *[%])	
Cardiovascular	79 (98.8)
Respiratory	72 (90.0)
Renal	32 (40.5)
Haematological	28 (35.0)
Metabolic acidosis	68 (85.0)
Number of organ systems failing during first 24 hours of stay in critical care (*n *[%])	
<2	1 (1.3)
2	5 (6.3)
3	38 (47.5)
4	26 (32.5)
5	10 (12.5)
Primary site of infection^d ^(*n *[%])	
Lung	20 (27.0)
Abdomen	33 (44.6)
Urinary tract	5 (6.8)
Other	16 (21.6)
Positive blood culture^e ^(*n *[%])	26 (36.1)
Organisms cultured (*n *[%])	
Any	52 (65.8)
Gram-negative	22 (42.3)
Gram-positive	26 (50.0)
Fungus	12 (23.1)
Mortality (*n *[%])	
Unit	31 (3 8.8)
Hospital^f^	39 (48.8)
Location at 28 days (*n *[%])	
Died	30 (37.5)
Unit	22 (27.5)
Hospital	22 (27.5)
Out of Hospital^f^	6 (7.5)
Unit length of stay (days; median [IQR])	
Unit survivors	20.7 (12.7–28.5)
Unit non-survivors	9.8 (5.9–23.9)
Hospital^g ^length of stay (days; median [IQR])	
Hospital survivors	60 (43–105)
Hospital nonsurvivors	19 (8–32)

^a^Other (source identified) was four from the oral/nasal cavity and two from the lungs/trachea. ^b^Evidence defined as follows, during prior 6 months (except those terms highlighted with asterisk, which are not restricted to the preceding 6 months): liver (biopsy proven cirrhosis, portal hypertension, hepatic encephalopathy); cardiovascular (very severe cardiovascular disease [New York Heart Association functional classification IV]); respiratory (severe respiratory disease [shortness of breath with light activity, for example walking 20 m on level ground], home ventilation); renal (chronic renal replacement therapy for irreversible renal disease); immunosuppressed (AIDS*, steroid treatment [daily], radiotherapy, chemotherapy, metastatic disease, acute myelogenous/lymphocytic leukaemia or multiple myeloma, chronic myelogenous/lymphocytic leukaemia, lymphoma, congenital immunohumoral or cellular immune deficiency state*). ^c^Surgical admissions are defined as those admitted directly to the unit from theatre and recovery. ^d^Primary site of infection was not reported for six (7.5%) admissions. ^e^A blood culture was reported as not done for six (7.5%) admissions. ^f^Discharged alive from acute hospital at or before 28 days. ^g^Excluding readmissions within the same hospital stay. APACHE, Acute Physiology and Chronic Health Evaluation; ICNARC, Intensive Care National Audit & Research Centre; IQR, interquartile range; SD, standard deviation.

### Nonrandomized comparison

Of patients receiving DrotAA, 1,079 (83.5%) met the definitions for severe sepsis and two or more organs failing during the first 24 hours, with 6.3% (0.4% to 27.4% across units) of all patients satisfying these definitions (Table 1). Individually matched control patients were successfully identified for between 609 (56.4%) and 922 (85.4%) patients receiving DrotAA, depending on control pool. Propensity-matched control patients were identified for between 818 (75.8%) and 1,053 (97.6%) patients. Both individual and propensity matching were successful in creating balance between cases and matched control patients on case mix factors, although balance was closer for individually matched control patients (Additional data file 3). The propensity model (Additional data file 4) had an area under the receiver operating characteristic curve of 0.79 and a pseudo-R^2 ^of 0.18.

**Figure 1:**
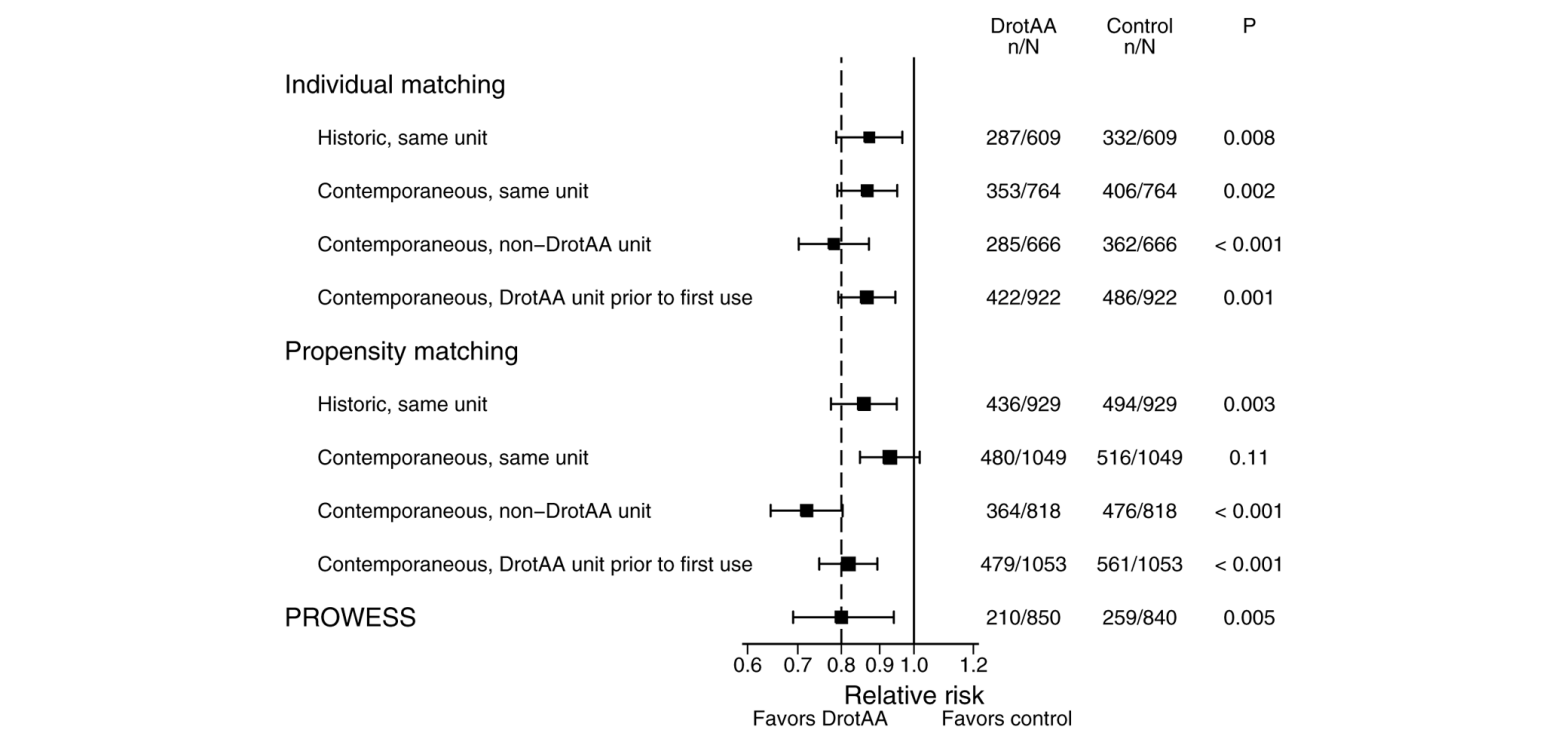
Primary results of matched cohort analyses on acute hospital mortality versus PROWESS at 28 days. PROWESS, Recombinant Human Activated Protein C Worldwide Evaluation in Severe Sepsis.

Relative risks (range 0.78 to 0.87) estimated from individually matched cohorts (on ultimate acute hospital mortality) were all consistent with PROWESS (on 28-day mortality; Figure 1). Relative risks from propensity-matched cohorts were more varied (range 0.72 to 0.93); in only one case did the 95% confidence interval exclude the PROWESS result and include the null value.

**Figure 2:**
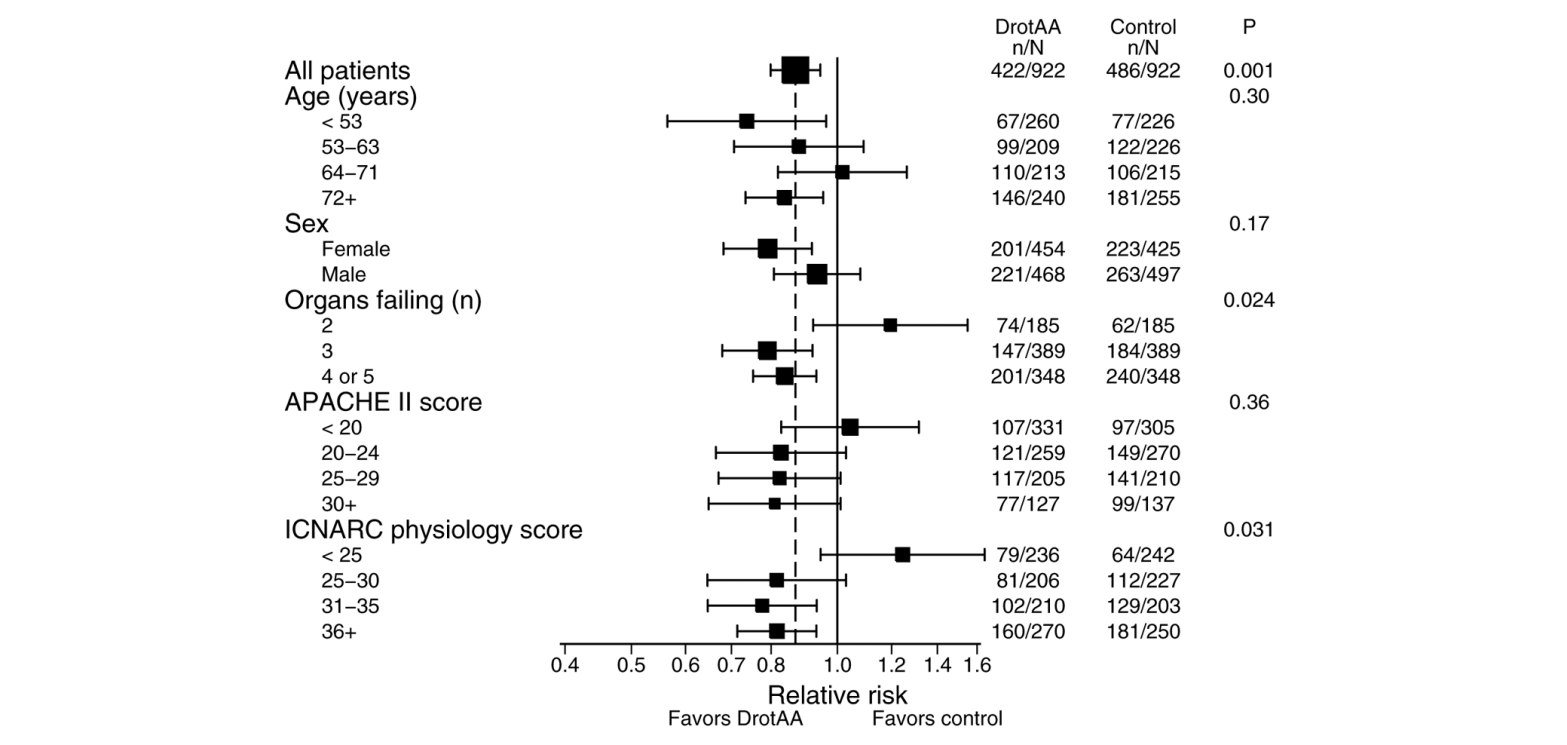
Subgroup results: acute hospital mortality for individual matching to control pool 4. Pool 4 includes patients from a contemporaneous drotrecogin alfa (activated; DrotAA) unit but before the first use in that unit.

All individually and propensity-matched results exhibited similar patterns across subgroups (control pool 4 [see numbered list above] presented in Figure 2). There were no significant differences in effectiveness across subgroups except for ICNARC physiology score and number of organs failing during the first 24 hours, which exhibited no effect for patients with lower severity disease and no effect for those with two organs failing, as compared with a significant reduction in mortality for thosse with three or more organs failing, respectively.

Restricting the analysis to patients receiving DrotAA during the first 24 hours in the unit resulted in larger observed treatment effects (relative risks 0.62 to 0.81). Restricting the anlaysis to matched pairs with the closest match on physiology did not change the results.

## Discussion

Overall, the rate of use of DrotAA, although increasing over time, appeared to be low and varied across the units studied, perhaps reflecting anecdotal evidence of uncertainty among UK clinicians. The rate of use of DrotAA was approximately one in 16 for admissions meeting the definitions for severe sepsis and two or more organ systems failing. Relative to those who did not receive DrotAA, patients receiving DrotAA were younger and more severely ill, as indicated either by percentage ventilated in the first 24 hours or by number of organ systems failing, but they were less likely to have serious conditions in their past medical history. For patients receiving DrotAA, almost one-third were reported as not receiving the full 96-hour DrotAA infusion, almost a quarter were reported as having an interrupted infusion, and 8% were reported as having a serious adverse event in the opinion of the responsible clinician.

Employing two different statistical approaches (individual case matching and propensity model matching) and using four different control pools, the nonrandomized comparison of the effectiveness of DrotAA on acute hospital mortality resulted in eight different point estimates, seven of which were consistent with the findings of PROWESS at 28 days. Subgroup analyses revealed that DrotAA was effective in patients with three or more organs failing, which again was consistent across both analytical approaches and all control pools.

These data are the first, rigorous data across a large, representative sample of UK critical care units to report real-life DrotAA use and outcomes. Although a representative sample of units participated, no overall UK DrotAA use data were available to us, and so we do not know whether higher users of DrotAA were included among nonparticipating units. The large sample size, for both units and patients, and the basis of actual use of DrotAA (not subject to entry criteria used in the PROWESS study) enhance the generalizability of these results. Selection for DrotAA was at the discretion of the responsible clinician in each unit. No investigation was conducted into the details of the protocols in place or decision-making process for use of DrotAA in the participating units. Control patients did not receive a directly comparable placebo infusion as in PROWESS, and therefore measurement of infusion-related outcome data was not possible. In addition, because they were not part of the routine outcome audit, primary site of infection, blood culture and adverse event data were only collected for those receiving DrotAA. Adverse event data were based on the opinion of the responsible clinician, as compared with the more stringent and robust definitions and assessment used in the PROWESS study. Further standardization of adverse event data was not attempted, and under-reporting or over-reporting might have occurred. Although longer term outcomes and health-related quality of life data are clearly important, reporting survival to acute hospital discharge (median 44 days) appeared to be an improvement over 28 days.

Nonrandomized studies are without doubt a second choice design for measuring treatment effects. Clearly, differences found between DrotAA and control groups cannot be attributed directly to DrotAA, but to DrotAA and any residual selection bias. Although sophisticated statistical techniques were used to attempt to create equality between the groups, existence of selection bias is not ruled out. Higher numbers matched are usually achieved with propensity matching (on a single estimate of likelihood) than individual matching (on a number of matching criteria). The lowest match in this study (56.4% for individual matching with historical control patients in the same unit) was hampered by the lack of historic CMP data (no control data for 10 of the units). In general, matches to control patients from the same unit were also inevitably less complete than those in which the control patients could come from multiple units. Despite this, both propensity and individual matching were successful in creating balance between cases and matched controls on case mix factors, although balance was closer for individually matched control patients.

Relative to PROWESS and ENHANCE (the Eli Lilly and Company sponsored, nonrandomized, open-label study) [13], the characteristics of patients receiving DrotAA in our study indicated that they were only slightly younger or similar in age (mean age 59 years versus 61 and 59, respectively), were more likely to have an abdominal infection (40% versus 20% and 25%) and were more severely ill, indicated either by receipt of ventilation (90% versus 73% and 82%) or by two or more organs failing (95% versus 75% and 84%). Other studies have yielded similar results [17-20]. Hospital mortality was much higher than in PROWESS (45% versus 29%) and, although overall serious adverse events were less frequent, the incidence of serious bleeding events was greater in our study than in PROWESS (6% versus 3.5%) and similar to that in ENHANCE (6.0% versus 6.5%).

Our finding that DrotAA appeared to be effective also concurs with the PROWESS data for acute hospital mortality (relative risk 0.85, 95% confidence interval 0.74 to 0.98) [21] and with the findings of a number of other, nonrandomized studies [17,20,22,23]. Our findings that DrotAA appeared not to be effective in patients with less severe disease (indicating no effect for lower ICNARC physiology scores and for patients with two organs failing, as compared with a significant reduction in mortality for higher scores and for patients with three or more organs failing) is consistent with the findings of a further Eli Lilly and Company sponsored, randomized controlled trial conducted in patients at low risk for death, which was stopped early because of futility [24].

The need for further debate regarding whether 28 days is the optimum end-point for studies in severe sepsis was reinforced by our study. That only 17.5% of patients were discharged from acute hospital (27.3% of all survivors at 28 days), similar to findings in the PROWESS study (42.0% of patients were discharged from acute hospital, 58.2% of all survivors at 28 days), supported the view that 28 days may be too early in the course of critical illness to power studies to measure benefit for patients with severe sepsis.

## Conclusion

The nonrandomized, matched analyses resulted in eight different point estimates, seven of which were consistent with the results of PROWESS. However, this study neither negates the need for new data from a randomized controlled trial nor contradicts the current UK National Institute for Health and Clinical Excellence guidelines for use of DrotAA. However, as soon as new randomized data are available, National Institute for Health and Clinical Excellence guidance should be revisited as a priority. The announcement of the new, Eli Lilly and Company funded randomized controlled trial is welcomed, and it is hoped that the investigators will include UK centres in this study and ensure that it is adequately powered to be able to detect differences in outcome beyond 28 days.

## Key messages

• The rate of use of DrotAA in the UK appears low and varies across critical care units.

• The nonrandomized evaluation of effectiveness of DrotAA on acute hospital mortality resulted in eight different point estimates, seven of which were consistent with the results of PROWESS.

• The need for further debate regarding whether 28 days is the optimum end-point for studies in severe sepsis was reinforced by our study

• Current guidelines for use of DrotAA should be revisited, as a priority, once new randomized data are available

## Abbreviations

CMP = Case Mix Programme; DrotAA = drotrecogin alfa (activated); ENHANCE = Extended Evaluation of Recombinant Activated Protein C; FDA = Food and Drug Administration; ICNARC = Intensive Care National Audit & Research Centre; PROWESS = Recombinant Human Activated Protein C Worldwide Evaluation in Severe Sepsis.

## Competing interests

ICNARC conducts paid for analyses for industry, including Eli Lilly and Company. All authors are employees of ICNARC.

## Authors' contributions

KR conceived the study. EN coordinated the study. CW and DH performed the statistical analyses. KR, DH and CW drafted the manuscript. All authors contributed to the design and interpretation of the study and critical revision of the manuscript, and have read and approved the final manuscript.

## Supplementary Material

Additional file 1 Data collection form completed for admissions that received DrotAA. Presented is the data collection form completed for patient admitted who received DrotAA. Click here for file

Additional file 2 Timeline of the audit of DrotAA and other key events. Shown is the timeline of the audit of DrotAA and other key events. Click here for file

Additional file 3 Balance between admissions receiving DrotAA and matched controls. Shown is the balance between patients admitted who received DrotAA and matched control patients. Click here for file

Additional file 4 Propensity model for use of DrotAA. Shown is the propensity model for use of DrotAA. Click here for file
