# Supplementary material for: Drotrecogin alfa (activated): real-life use and outcomes for the UK
Source: Crit Care. 2008 Apr 22;12(2):R58. doi: 10.1186/cc6879 (PMC2447613; doi:10.1186/cc6879)
Supplement: Additional file 1 — Data collection form completed for admissions that received DrotAA. Presented is the data collection form completed for patient admitted who received DrotAA. [file cc6879-S1.pdf]

Audit of the use of drotrecogin alfa (activated) for severe sepsis (Xigris)

case mix programme

TO Emma North
Research Administrator, ICNARC
Fax Number: 020 7388 3759

FROM

Name: ______________________

Hospital: ______________________

Today's date: ☐☐ ☐☐ ☐☐☐☐

ADMISSION

CMP Admission Number: ☐☐☐☐☐☐☐☐

Date of admission to your unit: ☐☐ ☐☐ ☐☐☐☐

INFUSION

Date of first infusion of Xigris: ☐☐ ☐☐ ☐☐☐☐

Time of first infusion of Xigris: ☐☐:☐☐

Did the patient receive the complete 96-hour infusion of Xigris? Yes ☐ No ☐

If **No**, please state why not:

____

____

Was there any interruption in the infusion of Xigris? Yes ☐ No ☐

If **Yes**, on what date and at what time did the interruption occur? Date: ☐☐ ☐☐ ☐☐☐☐ Time: ☐☐:☐☐

What was the duration of the interruption? (hours:mins) ☐☐:☐☐

What was the reason for the interruption in the infusion?

____

____

Was this infusion given as part of a study/trial? Yes ☐ No ☐ Name of study/trial? ______________________

INFECTION

Do you know the primary site of this patient's infection? Yes ☐ No ☐

If **Yes**, please indicate the site: Lung: ☐ Abdomen: ☐ Urinary tract: ☐ Other: ☐

If **Other**, please state site: ______________________

Was a blood culture done prior to the infusion of Xigris? Yes ☐ No ☐ ⇨ If **Yes**, was it positive? Yes ☐ No ☐

Was an organism cultured for this patient's infection? Yes ☐ No ☐

If **Yes**, please tick **all** organisms cultured:

Gram-negative bacteria ☐ Gram-positive bacteria ☐ Fungus ☐

Name of primary pathogen (or state if not known) ______________________

ADVERSE EVENTS

Did a serious adverse event occur (any adverse event assessed as serious by the clinician)? Yes ☐ No ☐

If **Yes**, was it a: Serious bleeding event §? Yes ☐ No ☐ Thrombotic event? Yes ☐ No ☐ Other event? Yes ☐ No ☐

If a **serious bleeding event**, please indicate site(s) :

If **Other event**, please state: ______________________

Gastrointestinal bleed: ☐ Genitourinary bleed: ☐ Skin or soft tissue bleed: ☐

Intrathoracic bleed: ☐ Intraabdominal bleed: ☐ Other (source unidentified) bleed: ☐

Intracranial bleed: ☐ Retroperitoneal bleed: ☐ Other (source identified) bleed: ☐

If **Other (source identified) bleed**, please state site(s): ______________________

§ Serious bleeding events are defined as any intracranial haemorrhage, any life-threatening bleed or any bleeding event requiring the administration of >= 3 units of packed red blood cells per day for two consecutive days, or any bleeding event assessed as serious by the clinician

© 2004

Intensive Care National Audit & Research Centre

All rights reserved.
