# Supplementary material for: Drotrecogin alfa (activated): real-life use and outcomes for the UK
Source: Crit Care. 2008 Apr 22;12(2):R58. doi: 10.1186/cc6879 (PMC2447613; doi:10.1186/cc6879)
Supplement: Additional file 2 — Timeline of the audit of DrotAA and other key events. Shown is the timeline of the audit of DrotAA and other key events. [file cc6879-S2.pdf]

Timeline of the audit of drotrecogin alfa (activated) and other key events

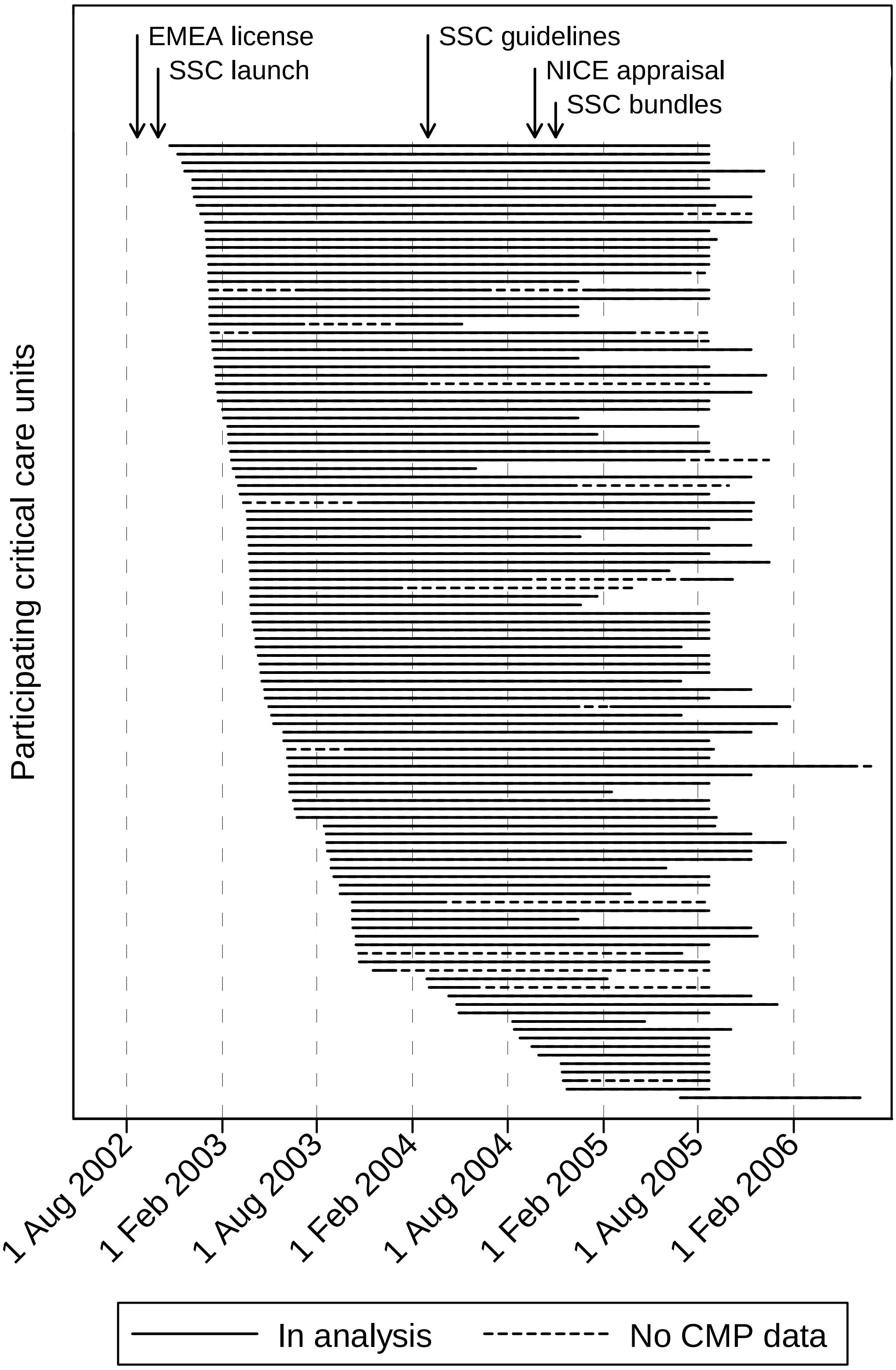

CMP, Case Mix Programme; EMEA, European Agency for the Evaluation of Medicinal Products; NICE, National Institute for Health and Clinical Excellence; SSC, Surviving Sepsis Campaign.
