# Supplementary material for: Drotrecogin alfa (activated): real-life use and outcomes for the UK
Source: Crit Care. 2008 Apr 22;12(2):R58. doi: 10.1186/cc6879 (PMC2447613; doi:10.1186/cc6879)
Supplement: Additional file 3 — Balance between admissions receiving DrotAA and matched controls. Shown is the balance between patients admitted who received DrotAA and matched control patients. [file cc6879-S3.pdf]

Balance between admissions receiving drotrecogin alfa (activated) (DrotAA) and matched controls

Control pool		(a) Historic, same unit		(b) Contemporaneous, same unit		(c) Contemporaneous, non-DrotAA unit		(d) Contemporaneous, DrotAA unit prior to first use	
**Matched group**		**DrotAA**	**Control**	**DrotAA**	**Control**	**DrotAA**	**Control**	**DrotAA**	**Control**
Individual matching		***N=609***	***N=609***	***N=764***	***N=764***	***N=666***	***N=666***	***N=922***	***N=922***
Age[a], mean (SD)		61.7 (14.0)	62.1 (14.0)	61.0 (14.9)	61.8 (14.9)	59.4 (15.2)	59.6 (15.2)	60.0 (15.4)	60.9 (15.0)
Sex, n (%)	Female	299 (49.1)	280 (46.0)	369 (48.3)	359 (47.0)	324 (48.6)	330 (49.5)	454 (49.2)	425 (46.1)
	Male	310 (50.9)	329 (54.0)	395 (51.7)	405 (53.0)	342 (51.4)	336 (50.5)	468 (50.8)	497 (53.9)
Ventilated[b], n (%)		572 (93.9)	554 (91.0)	712 (93.2)	663 (86.8)	620 (93.1)	555 (83.3)	858 (93.1)	805 (87.3)
Number of organ systems failing[b;c], n (%)	2	142 (23.3)	142 (23.3)	167 (21.9)	167 (21.9)	139 (20.9)	139 (20.9)	185 (20.1)	185 (20.1)
	3	262 (43.0)	262 (43.0)	339 (44.4)	339 (44.4)	289 (43.4)	289 (43.4)	389 (42.2)	389 (42.2)
	4	174 (28.6)	174 (28.6)	229 (30.0)	229 (30.0)	205 (30.8)	205 (30.8)	285 (30.9)	285 (30.9)
	5	31 (5.1)	31 (5.1)	29 (3.8)	29 (3.8)	33 (5.0)	33 (5.0)	63 (6.8)	63 (6.8)
APACHE II, mean (SD)	APS	18.1 (5.8)	18.1 (6.2)	18.2 (6.0)	17.5 (6.2)	18.4 (6.3)	19.2 (6.6)	18.4 (6.2)	18.2 (6.0)
	Score	22.3 (6.3)	22.6 (6.8)	22.3 (6.5)	22.0 (6.8)	22.3 (6.7)	23.3 (7.3)	22.4 (6.6)	22.6 (6.8)
ICNARC physiology score[1], mean (SD)		29.4 (7.9)	28.8 (7.4)	29.3 (7.7)	28.5 (7.7)	29.5 (7.8)	29.0 (7.5)	29.8 (8.1)	29.4 (7.7)
Propensity matching		***N=929***	***N=929***	***N=1,049***	***N=1,049***	***N=818***	***N=818***	***N=1,053***	***N=1,053***
Age[d], mean (SD)		59.0 (16.2)	58.3 (16.7)	58.5 (16.2)	57.4 (16.1)	58.3 (16.2)	57.6 (15.8)	58.6 (16.2)	58.3 (15.5)
Sex[d], n (%)	Female	465 (50.1)	484 (52.1)	520 (49.6)	531 (50.6)	400 (48.9)	448 (54.8)	524 (49.8)	543 (51.6)
	Male	464 (49.9)	445 (47.9)	529 (50.4)	518 (49.4)	418 (51.1)	370 (45.2)	529 (50.2)	510 (48.4)
Ventilated[b], n (%)		864 (93.0)	850 (91.5)	970 (92.5)	930 (88.7)	758 (92.7)	665 (81.3)	974 (92.5)	926 (87.9)
Number of organ systems failing[b;d], n (%)	2	173 (18.6)	206 (22.2)	193 (18.4)	210 (20.0)	148 (18.1)	136 (16.6)	193 (18.3)	187 (17.8)
	3	361 (38.9)	370 (39.8)	418 (39.8)	436 (41.6)	329 (40.2)	351 (42.9)	421 (40.0)	457 (43.4)
	4	314 (33.8)	280 (30.1)	352 (33.6)	317 (30.2)	272 (33.3)	273 (33.4)	356 (33.8)	289 (27.4)
	5	81 (8.7)	73 (7.9)	86 (8.2)	86 (8.2)	69 (8.4)	58 (7.1)	83 (7.9)	120 (11.4)
APACHE II, mean (SD)	APS	18.9 (6.3)	18.3 (6.2)	18.8 (6.4)	18.2 (6.3)	18.8 (6.5)	20.0 (6.8)	18.8 (6.4)	17.8 (5.6)
	Score	22.8 (6.7)	21.9 (6.6)	22.6 (6.7)	21.9 (6.8)	22.6 (6.9)	23.7 (6.9)	22.7 (6.8)	21.6 (6.1)
ICNARC physiology score[d], mean (SD)		30.5 (8.4)	29.6 (7.8)	30.4 (8.4)	29.7 (8.1)	30.3 (8.2)	29.6 (7.8)	30.4 (8.4)	29.5 (7.6)

APACHE: Acute Physiology And Chronic Health Evaluation; APS: acute physiology score; ICNARC: Intensive Care National Audit & Research Centre; SD: standard deviation

[a] Included in matching procedure

[b] During first 24 hours in the critical care unit

[c] Perfect balance ensured by matching procedure

[d] Included in propensity model
