# Supplementary material for: Drotrecogin alfa (activated): real-life use and outcomes for the UK
Source: Crit Care. 2008 Apr 22;12(2):R58. doi: 10.1186/cc6879 (PMC2447613; doi:10.1186/cc6879)

Propensity model for use of drotrecogin alfa (activated)

Factors related to the decision to use DrotAA		Odds ratio (95% CI)	p-value
Unit/time factors			
Local approval for DrotAA		3.78 (2.67 – 5.34)	< 0.001
NICE appraisal		0.92 (0.79 – 1.08)	0.306
Hospital type	University	1	0.063
	University affiliated	0.96 (0.67 – 1.40)	
	Non-university	0.66 (0.48 – 0.90)	
Number of beds in unit		0.96 (0.92 – 1.01)	0.132
Patient factors			
Age		Non-linear*	< 0.001
ICNARC physiology score		Non-linear*	< 0.001
Sex (male)		0.84 (0.73 – 0.96)	0.009
Source	A&E or other hospital	1	0.006
	ICU or HDU	1.00 (0.78 – 1.29)	
	Theatre – elective	0.88 (0.55 – 1.40)	
	Theatre – emergency	1.26 (0.90 – 1.77)	
	Ward or intermediate care area	0.77 (0.64 – 0.93)	
Organ systems failing	Cardiovascular	4.44 (2.26 – 8.72)	< 0.001
	Respiratory	1.89 (1.45 – 2.45)	< 0.001
	Renal	1.08 (0.91 – 1.27)	0.372
	Haematological	0.91 (0.76 – 1.10)	0.334
	Metabolic acidosis	2.22 (1.86 – 2.66)	< 0.001
Severe chronic past medical history	Liver	0.19 (0.09 – 0.37)	< 0.001
	Cardiovascular	0.28 (0.11 – 0.71)	0.007
	Respiratory	0.54 (0.34 – 0.85)	0.008
	Renal	0.32 (0.17 – 0.58)	< 0.001
	Immuno-compromised	0.59 (0.45 – 0.76)	< 0.001
Primary reason for admission	Cardiovascular	1	< 0.001
	Respiratory (non-surgical)	0.89 (0.72 – 1.09)	
	Respiratory (surgical)	0.73 (0.40 – 1.34)	
	Neurological (non-surgical)	0.58 (0.37 – 0.91)	
	Neurological (surgical)	0.29 (0.04 – 2.17)	
	Gastrointestinal (non-surgical)	0.80 (0.59 – 1.09)	
	Gastrointestinal (surgical)	0.74 (0.54 – 1.02)	
	Genitourinary (non-surgical)	0.71 (0.49 – 1.02)	
	Genitourinary (surgical)	0.82 (0.39 – 1.72)	
	Endocrine	0.18 (0.08 – 0.40)	
	Haematological (non-surgical)	1.89 (1.41 – 2.55)	
	Haematological (surgical)	1.93 (0.96 – 3.88)	
	Musculoskeletal (non-surgical)	0.17 (0.02 – 1.25)	
	Musculoskeletal (surgical)	0.37 (0.08 – 1.63)	
	Dermatological (non-surgical)	0.55 (0.16 – 1.88)	
	Dermatological (surgical)	1.00 (0.41 – 2.41)	

* Fitted with restricted cubic splines – see separate plots

Non-linear relationships for age and ICNARC physiology score

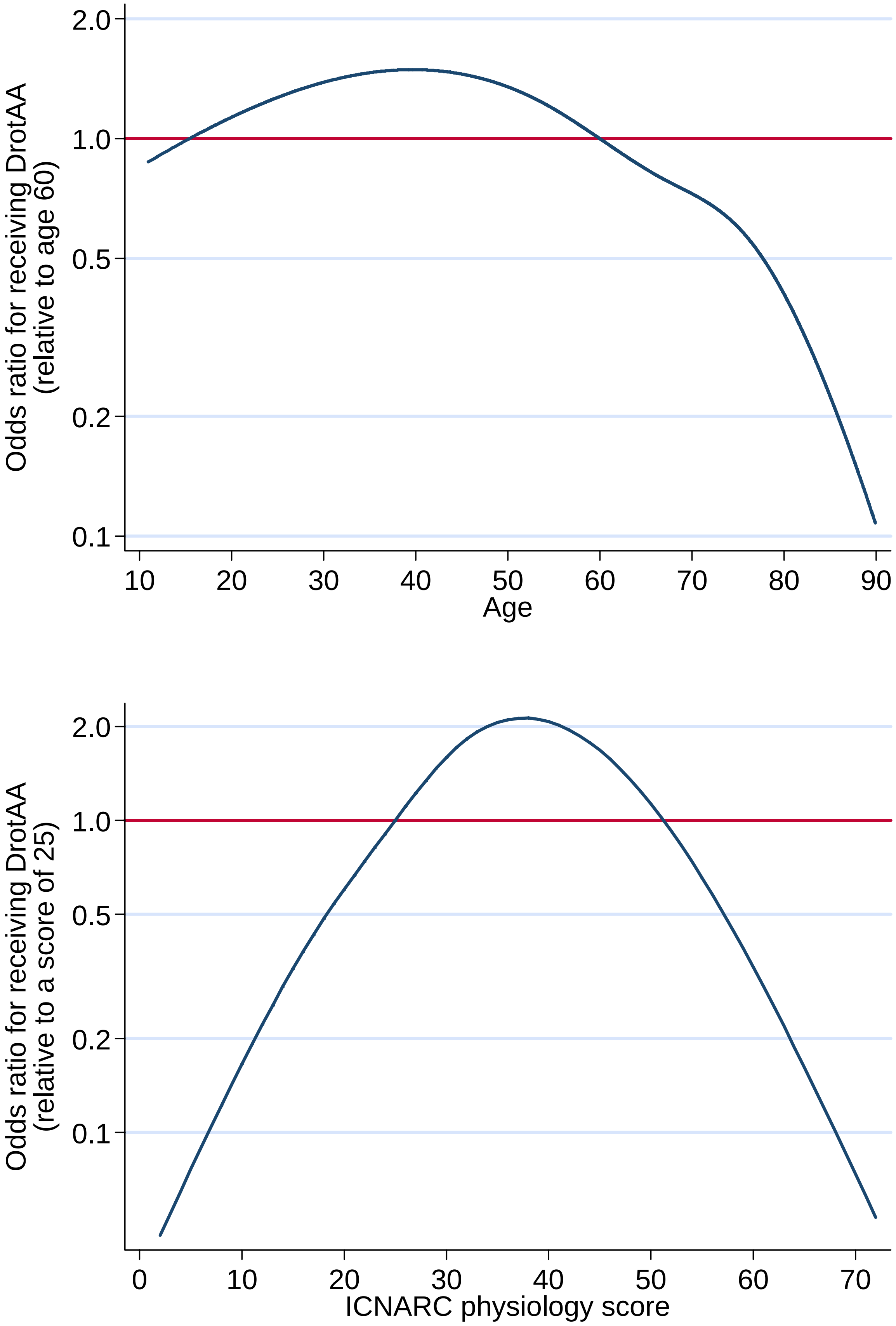

Supplement: Additional file 4 — Propensity model for use of DrotAA. Shown is the propensity model for use of DrotAA. [file cc6879-S4.pdf]
